# Prophylactic Appendectomy during Laparoscopic Surgery for Other Conditions

**DOI:** 10.1155/2014/292864

**Published:** 2014-07-20

**Authors:** S. Occhionorelli, R. Stano, S. Targa, S. Maccatrozzo, L. Cappellari, G. Vasquez

**Affiliations:** Arcispedale Sant'Anna, Department of Surgery, Emergency Surgery Service, Via Aldo Moro 8, Cona, 44124 Ferrara, Italy

## Abstract

Acute appendicitis remains the most common surgical emergency. Laparoscopy has gained increasing favor as a method of both investigating right iliac fossa pain and treating the finding of appendicitis. A question arises: what to do with an apparent healthy appendix discovered during laparoscopic surgery for other pathology. We present a case of unilateral hydroureteronephrosis complicated with rupture of the renal pelvis, due to gangrenous appendicitis with abscess of the right iliopsoas muscle and periappendicular inflammation in a 67-year-old woman, who underwent laparoscopic right annessiectomy for right ovarian cyst few years earlier, in which a healthy appendix was left inside. There is a lack of consensus in the literature about what to do with a normal appendix. The main argument for removing an apparently normal appendix is that endoluminal appendicitis may not be recognized during surgery, leading to concern that an abnormal appendix is left in place. Because of a lack of evidence from randomized trials, it remains unclear whether the benefits of routine elective coincidental appendectomy outweigh the costs and risks of morbidity associated with this prophylactic procedure. Nevertheless, it appears, from limited data, that women aged 35 years and under benefit most from elective coincidental appendectomy.

## 1. Introduction

Acute appendicitis remains the most common surgical emergency and although diagnosis should be made on clinical grounds, sometimes this can be difficult. Laparoscopy has gained increasing favor as method of both investigating right iliac fossa pain and treating the finding of appendicitis. Hydroureteronephrosis can be a sign of presentation of acute appendicitis, even though it is not as common as pain, nausea, vomiting, and fever [1, 2]. If we add the rupture of the renal pelvis and abscess of the iliopsoas muscle to the pool of symptoms, it is understandable that we are dealing with a rare and complex presentation of acute appendicitis.

## 2. Patients and Methods

A 67-year-old woman presented with a three-day history of growing right lower quadrant (RLQ) abdominal pain. The pain was referred to be in right iliac region, radiating to right lumbar region. No macroscopic haematuria was present.

The patient history recorded an essential thrombocytosis, a previous radical mastectomy followed by radiochemotherapy for breast cancer, removal of cutaneous recurrence of breast cancer followed by chemotherapy, and a laparoscopic right annessiectomy for right ovarian cyst few years before.

The patient described the pain as a worsening continuous ache with sudden onset in right iliac fossa and right flank. Physical examination revealed a distended abdomen; deep palpation was pain evoking in RLQ with signs of peritoneal irritation (the Blumberg sign was considered slightly positive). Percussion revealed bowel tympany in all abdominal quadrants and the auscultation revealed no change in bowel movement pattern.

Blood test was significant for infection: white blood cells count was 24,100/mm³ with more than 90% neutrophils; C reactive protein was 22,56 mg/dL; haemoglobin level was normal (13,5 g/dL; hematocrit 42%) and renal function was preserved: 1,3 mg/dL.

**Figure 1:**
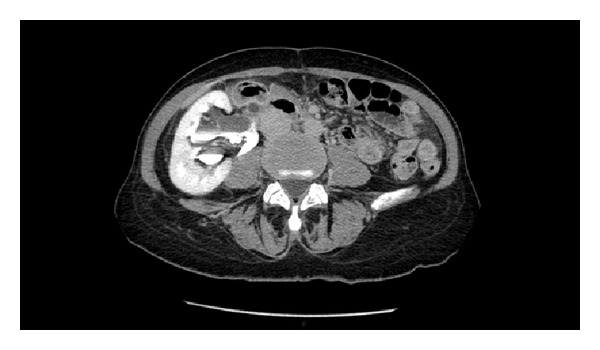
Right hydroureteronephrosis with delay in opacification of right urinary system.

**Figure 2:**
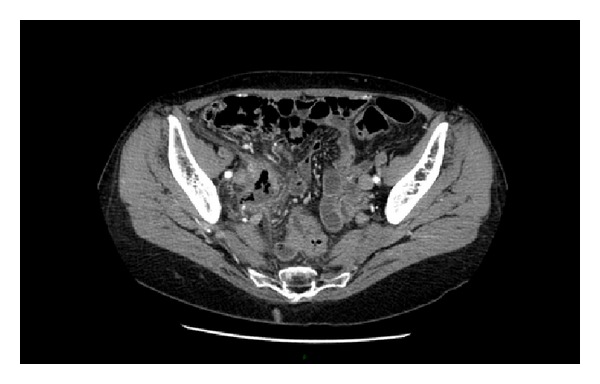
Pelvic mass involving right ureter.

Abdominal ultrasonography demonstrated right renal pelvis dilatation and perirenal fluid film at lower pole of right kidney. Abdominal CT scan with contrast media injection (Figure 1) revealed right ureter of enlarged diameter throughout its course, with abrupt stop after crossing right iliac vessels (Figure 2) where it seems to be involved in an aggregate of intestinal loops. Delayed and incomplete opacification of the right urinary system after 15 minutes suggested either the presence of a ureteral duplication or the extravasation of iodinated contrast medium.

After urological evaluation, a right lumbar percutaneous pyelography, demonstrating perirenal contrast extravasation due to possible pelvic lesion, was performed. Right kidney appears to be ptotic and malrotated. Soon percutaneous nephrostomy was made leading to pain relief.

Considering clinical symptoms, lab tests, and instrumental evaluations, urgent explorative laparotomy was decided. First, ascendent pyelography was performed. Abrupt stop of pelvic ureter was pointed out: a metallic stent was positioned in order to identify the ureter during plans dissection and to avoid its accidental lesion.

At the time of the operation the appendix appeared to be retrocecal and gangrenous, surrounded by a periappendicular abscess involving and compressing right ureter from the outside. The tip of the appendix, completely necrotic, was in touch with right iliopsoas muscle, creating an abscess.

The patient underwent open appendectomy and minimal resection of distal ileus and caecum due to spreading of the inflammation to bowel tissues.

On postoperative day 4, a radiographic control with percutaneous nephrostomic injection of contrast medium was made, revealing opacification of urinary bladder and well-positioned ureteral stent, so percutaneous nephrostomy was removed. The patient was discharged 7 days after surgery. One month later, ultrasound abdominal control revealed no kidney complications and regular presence of urinary stent, which was (then) removed.

## 3. Discussion

If the patient underwent coincidental appendectomy, at the time of previous intervention of laparoscopic right annessiectomy, she would not have developed appendicitis and its complications.

Appendectomy is a simple and well-standardized surgery [3]; it can be made open or laparoscopic. The laparoscopic way seems to be superior to open approach in terms of pain, wound infection rate and postoperative ileus [3, 4]. It is well known the role of laparoscopic appendectomy in making gynecological diagnosis (diagnosis of gynecological condition is made in 73% of cases during laparoscopic appendectomy versus 17% of cases during open appendectomy) and this is because laparoscopy affords a more complete vision of the deep pelvis [5], but the role of prophylactic appendectomy during laparoscopic surgery for other abdominal conditions (e.g., gynecological surgery) remains unclear.

There is no consensus about what to do with “healthy” appendix. Some authors suggest that normal-looking appendices should be removed [3, 6] during laparoscopy for acute right iliac fossa pain, whereas other authors alert surgeons to the life-threatening consequences of performing a “healthy appendectomy” [7, 8], such as trauma induced by anaesthesia and surgery, higher rate of infectious complications.

Because of a lack of evidence from randomized trials [9], related to the fact that risk-benefit analysis varies according to patient's age and history, the decision to perform an elective coincidental appendectomy at the time of operation for other unrelated surgical conditions should be based on individual clinical scenarios and patient's characteristics. Nevertheless it appears, from limited data, that women aged 35 years and under benefit most from elective coincidental appendectomy [9]. Most studies suggest that there is little, if any, increased morbidity associated with elective coincidental appendectomy at the time of gynaecological surgery, whether performed during open surgical procedure or during laparoscopy [9]. Cases of symptoms requiring reoperation for appendectomy have been described in patients whose normal appendix was left in place at the time of the original procedure (as in our case) [4]. Even in lack of evidence, gynecologists seem to be largely geared towards removing the appendix during surgery for other gynecological pathologies although this appears to be normal [9, 10]; furthermore, intraoperative diagnosis is not easy, with almost one-third of apparently normal appendices being inflamed histologically [6, 11, 12].

## 4. Conclusion

Since our experience in prophylactic appendectomy during laparoscopic surgery for other conditions demonstrates few complications, we prefer removing the appendix in order to avoid unfortunate consequences, such as the case we had presented. The risk of morbidity associated with prophylactic procedure is very low but the benefit of coincidental appendectomy remains controversial and is still open to debate. Nevertheless it appears, from limited data, that women aged 35 years and under benefit most from elective coincidental appendectomy.
